# Exploring why European primary care physicians sometimes do not think of, or act on, a possible cancer diagnosis. A qualitative study

**DOI:** 10.3399/BJGPO.2023.0029

**Published:** 2023-09-20

**Authors:** Senada Hajdarevic, Cecilia Högberg, Mercè Marzo-Castillejo, Vija Siliņa, Jolanta Sawicka-Powierza, Magadalena Esteva, Tuomas Koskela, Davorina Petek, Sara Contreras-Martos, Marcello Mangione, Zlata Ožvačić Adžić, Radost Asenova, Svjetlana Gašparović Babić, Mette Brekke, Krzysztof Buczkowski, Nicola Buono, Saliha Serap Çifçili, Geert-Jan Dinant, Babette Doorn, Robert D Hoffman, George Kuodza, Peter Murchie, Liina Pilv, Aida Puia, Aurimas Rapalavicius, Emmanouil Smyrnakis, Birgitta Weltermann, Michael Harris

**Affiliations:** 1 Department of Nursing, Umeå University, Umeå, Sweden; 2 Department of Public Health and Clinical Medicine, Family Medicine, Umeå University, Umeå, Sweden; 3 Department of Public Health and Clinical Medicine, Education and Development Östersund, Unit of Research, Umeå University, Umeå, Sweden; 4 Research Support Unit Metropolitana Sud, University Institute for Primary Health Care Research IDIAPJordi Gol, Catalan Health Institute, Barcelona, Spain; 5 Department of Family Medicine, Riga Stradiņš University, Riga, Latvia; 6 Department of Family Medicine, Medical University of Białystok, Białystok, Poland; 7 Majorca Primary Care Department, Spain; 8 Balearic Islands Health Research Institute (IdISBa), Balearic Islands, Spain; 9 Faculty of Medicine and Health Technology, Tampere University, Tampere, Finland; 10 Center of General Practice,Tampere University Hospital, Tampere, Finland; 11 Department of Family Medicine, Faculty of Medicine, University of Ljubljana, Ljubljana, Slovenia; 12 Research Support Unit Metropolitana Sud, University Institute for Primary Health Care Research IDIAPJordi Gol, Catalan Health Institute, Barcelona, Spain; 13 Local Health Authority Committee, Palermo City, Italy; 14 Department of Family Medicine, University of Zagreb, School of Medicine, Zagreb, Croatia; 15 Health Center Zagreb-Centar, Zagreb, Croatia; 16 Department Urology and General Practice, Medical University of Plovdiv, Plovdiv, Bulgaria; 17 Croatian Health Insurance Fund, Rijeka, Croatia; 18 Department of Health and Society, General Practice Research Unit, University of Oslo, Oslo, Norway; 19 Nicolaus Copernicus University, Toruń, Poland; 20 Department of General Practice, National Society of Medical Education in General Practice (SNaMID), Caserta, Italy; 21 Family Medicine Department, Marmara University Medical School, Istanbul, Turkey; 22 Department of General Practice, Maastricht University, Maastricht, The Netherlands; 23 Department of Family Medicine, Sackler Faculty of Medicine, Tel Aviv University, Tel Aviv, Israel; 24 Department of Family Medicine, Maccabi Healthcare Services, Southern District, Israel; 25 Department of Family Medicine and Outpatient Care, Medical Faculty #2, Uzhhorod National University, Uzhgorod, Ukraine; 26 Centre of Academic Primary Care, Institute of Applied Health Sciences, University of Aberdeen, Aberdeen, UK; 27 Institute of Family Medicine and Public Health, University of Tartu, Tartu, Estonia; 28 Department of Family Medicine, University of Medicine and Pharmacy, Cluj-Napoca, Romania; 29 Family Medicine Department, Lithuanian University of Health Sciences, Kaunas, Lithuania; 30 Laboratory of Primary Health Care, General Practice and Health Services Research, Aristotle University of Thessaloniki, Thessaloniki, Greece; 31 Institut für Hausarztmedizin, University of Bonn, Bonn, Germany; 32 Institute of Primary Health Care Bern (BIHAM), University of Bern, Bern, Switzerland; 33 College of Medicine & Health, University of Exeter, Exeter, UK

**Keywords:** primary health care, physicians, primary care, cancer, Europe, diagnostic errors, qualitative research

## Abstract

**Background:**

While primary care physicians (PCPs) play a key role in cancer detection, they can find cancer diagnosis challenging, and some patients have considerable delays between presentation and onward referral.

**Aim:**

To explore European PCPs’ experiences and views on cases where they considered that they had been slow to think of, or act on, a possible cancer diagnosis.

**Design & setting:**

A multicentre European qualitative study, based on an online survey with open-ended questions, asking PCPs for their narratives about cases when they had missed a diagnosis of cancer.

**Method:**

Using maximum variation sampling, PCPs in 23 European countries were asked to describe what happened in a case where they were slow to think of a cancer diagnosis, and for their views on why it happened. Thematic analysis was used to analyse the data.

**Results:**

A total of 158 PCPs completed the questionnaire. The main themes were as follows: patients’ descriptions did not suggest cancer; distracting factors reduced PCPs’ cancer suspicions; patients’ hesitancy delayed the diagnosis; system factors not facilitating timely diagnosis; PCPs felt that they had acted wrongly; and problems with communicating adequately.

**Conclusion:**

The study identified six overarching themes that need to be addressed. Doing so should reduce morbidity and mortality in the small proportion of patients who have a significant, avoidable delay in their cancer diagnosis. The ‘Swiss cheese’ model of accident causation showed how the themes related to each other.

## How this fits in

Few studies have focused on PCPs’ views, but they can find cancer diagnosis challenging, and some patients have considerable delays between presentation and onward referral. In this study, when asked to describe what happened in such cases, PCPs described a variety of issues, often with many such factors in a single case. The ‘Swiss cheese’ model can be used to understand how these failures relate to each other.

## Introduction

PCPs play a key role in cancer detection.^1,2^ However, cancer diagnosis can be challenging in primary care, as PCPs often see patients with non-specific symptoms that, while they could be due to cancer, are more often caused by benign conditions.^3,4^ Early diagnosis of cancer can be a difficult task, requiring knowledge and clinical experience,^5,6^ and challenging decisions on referral.^7^ System factors influence how quickly PCPs refer patients. These vary between the European healthcare systems,^8,9^ and can even vary within a healthcare system owing to differences in patient demographics and deprivation levels.^10^

Many patients with cancer are referred promptly by their PCPs, although some have considerable delays; for example, in a UK study, 8.3% of patients were still unreferred 90 days after presentation.^11^ Patients who experience referral delays are likely to have longer diagnostic intervals^12^ and poorer cancer survival rates.^13^ Cancer is one of the conditions that dominates diagnostic error reports from primary care.^7,14^

It is known that there can be missed opportunities to diagnose cancer in several phases of the diagnostic process,^8^ but few studies have focused on PCPs’ views. While one study explored European PCPs’ views on how cancer diagnoses could be made in a more timely way,^15^ another found that rural PCPs throughout Europe perceive greater cost, travel, and access barriers for their patients than their urban colleagues,^16^ and a Swedish study emphasised the challenges that PCPs face in sifting various symptoms and matching these to specific standardised cancer patient pathways.^17^ There is a gap in the research on PCPs’ own experiences of missing cancer diagnoses.

This study explored European PCPs’ experiences and views on particular cases where they considered that they had been slow to think of, or act on, a possible cancer diagnosis.

## Method

### Study design

A multicentre European qualitative study was undertaken, based on an online survey with open-ended questions which asked PCPs for their narratives about cases when they had been slow to think of a diagnosis of cancer.

### Development of the questionnaire

The Örenäs Research Group (ÖRG) is a European group of primary care researchers that studies the primary care factors that relate to cancer survival. A core group of ÖRG members designed a pilot questionnaire that was completed by 14 PCPs. The final text of the survey included an invitation for a narrative: *'Please write a short description of a time when you were slow to think of a cancer diagnosis, or where you thought of cancer but were slow to do something about it*.', followed by three free-text questions: *'What happened?', 'Why do you think it happened?',* and *'If you saw this patient presenting in the same way today, what would you do differently*?'. This article analyses PCPs’ replies to the first two of these questions.

### Participants and recruitment

Participants were GPs and doctors who had other specialist training but worked in the community and could be accessed directly by patients without referral.

ÖRG members from 23 countries (‘local leads’) helped to recruit PCPs from each country. To achieve maximum variation,^18^ the study purposefully included a balance of female and male PCPs, a range of years of experience, and different practice locations (rural and non-rural). Consent was implied by agreeing to take part in the survey.

### Data collection

Participants were sent a link to the online survey. To avoid the possibility that the meaning of the questions could change if translated, the survey questions were in English for all participants. Participants could answer the questions either in their own languages or, if they felt confident to do so, in English.

PCPs’ demographic data concerning country, gender, whether they were a trainee, years of working experience (≤4 years, 5–14 years, ≥15 years), and practice setting (town or city, rural, island or remote, or mixed) were collected. Answers in native languages were translated into English either by professional translators or by translators whose native language was English. Data were collected between December 2020 and April 2021.

### Analysis of data

Thematic analysis was used,^19,20^ an approach in which codes and themes are suggested by the data rather than by a theoretical framework. The phases of analysis included coding, followed by the identification and clustering of themes and sub-themes, and the production of a descriptive thematic summary. There was considerable overlap between PCPs’ responses to the questions *'What happened*?' and '*Why do you think it happened?*', so the data from these were combined before analysis. To manage the high volume of data, the core study group was divided into three subgroups. The researchers independently coded their subgroup’s 53 randomly assigned participants’ responses then compared them. Differences in researchers’ codes were discussed, refined, and resolved in online meetings. The data were then organised into themes and sub-themes in multiple online meetings at which they were discussed and agreed.

## Results

In total, 158 PCPs from 23 European countries submitted case descriptions and reflections (Table 1). More than half had at least 15 years of work experience, and just under one-quarter were PCP trainees. One-third of the responders worked in rural or mixed areas.

**Table 1.** Characteristics of the participants in the survey

Characteristic		Frequency, *n* (%)
*Total participants*		*158 (100)*
Gender	Female	89 (56.3)
	Male	68 (43.0)
	Prefer not to say	1 (0.6)
Work experience	≤4 years	15 (9.5)
	5–14 years	46 (29.1)
	≥15 years	97 (61.4)
Training status	Established PCP	121 (76.6)
	Trainee PCP	37 (23.4)
Area of work	Town or city	99 (62.7)
	Rural	33 (20.9)
	Island or remote	5 (3.2)
	Mixed	20 (12.6)
	Prefer not to say	1 (0.6)
Country	Bulgaria	10 (6.3)
	Croatia	8 (5.1)
	England	7 (4.4)
	Estonia	6 (3.8)
	Finland	5 (3.2)
	Germany	5 (3.2)
	Greece	8 (5.1)
	Ireland	10 (6.3)
	Israel	4 (2.5)
	Italy	10 (6.3)
	Latvia	10 (6.3)
	Lithuania	6 (3.8)
	The Netherlands	3 (1.9)
	Norway	7 (4.4)
	Poland	7 (4.4)
	Romania	8 (5.1)
	Scotland	5 (3.2)
	Slovenia	6 (3.8)
	Spain	14 (8.9)
	Sweden	8 (5.1)
	Switzerland	3 (1.9)
	Turkey	6 (3.8)
	Ukraine	2 (1.3)

PCP = primary care physician.

The analysis resulted in six themes, each with several sub-themes (Table 2). Many cases contained data belonging to several different themes. The themes and sub-themes are described below, with each quotation identified by participant number and country code.

**Table 2.** Themes and sub-themes

Theme	Sub-theme
Patients’ descriptions did not suggest cancer.	No ‘red flag’ symptoms or signs.Symptoms typical of common non-malignant conditions.Patients’ views or explanations were misleading.Patients said very little.
Distracting factors reduced PCPs’ cancer suspicions.	Cancer risk perceived as low.Investigations appeared to confirm a benign diagnosis.The cancers were rare ones.Improvement from symptomatic treatment.The patients had had similar symptoms in the past.Patients were frequent attenders.Other health issues dominated or confused.Impact from other people accompanying the patients.
Patients’ hesitancy delayed the diagnosis.	Primary care organisational factors made patients delay their presentations.Patients postponed follow-up.Patients’ social issues distracted from diagnostic process.
System factors not facilitating timely diagnosis.	High workloads.Long waits for tests or specialist opinions.Weaknesses in follow-up systems.Limited PCP access to diagnostic tools and specialist consultations.Gaps of continuity in primary care.Unclear responsibilities and poor interaction with secondary care.Lack of PCP experience and trainee supervision.
PCPs felt they had acted wrongly.	Poor or inadequate history-taking or physical examination.Too focused on specific symptoms or single possible diagnosis (‘tunnel thinking’).Delay in investigating or referring.Not noticing or not acting on abnormal test results.Follow-up not planned or forgotten.Trusting reassuring specialist opinions.Not taking patients seriously enough.Adapting to/accepting patients’ ideas about the diagnosis.
Problems with communicating adequately.	Not giving enough explanation to patients or being assertive enough with them.Reluctance to worry the patients.Unusual dynamics in patient–doctor relationships.Poor communication with secondary care and other healthcare providers.

PCP = primary care physician

### Patients’ descriptions did not suggest cancer

Some PCPs explained why they had not interpreted their patients’ stories and presenting symptoms as being indicative of cancer. They wrote that their patients’ descriptions did not raise any suspicion of cancer, or that they noticed no ‘red flags’ suggesting cancer. Some patients’ symptoms were interpreted as being typical of common non-malignant conditions:

*'I received a call from a patient who was complaining of a sore throat and a pain in the neck. It was the time of COVID pandemic, so my first thought was that he had some kind of infectious disease with respiratory symptoms* […] *my findings were compatible with the diagnosis of the respiratory infection.'* (112 /C)

Patients’ explanations could be misleading if they were related to other conditions. Some patients did not talk much about their symptoms or did not persist in telling the doctor about them:

'*Well, my thought is that I, as GP, after some time just stopped investigating thoroughly because I thought it went away — the patient stopped complaining. I think she thought that she was annoying me with her complaints since all those tests were normal.'* (82 /A)

### Distracting factors reduced PCPs’ cancer suspicions

Many factors could distract PCPs from considering cancer. The risk of cancer was sometimes perceived as low. Laboratory tests and physical examinations that were normal, or that appeared to confirm another diagnosis, reduced cancer suspicions, as could having symptoms from a rare cancer. Symptomatic improvement could also be a factor:

*'My patient was a 36-year-old woman with anaemia. Her only complaint was weakness. I done* [did a] *CBC* [blood count] *— found HGB* [haemoglobin] *98, prescribed her* [iron] *Fe 160 mg a day. After a month her HGB rose to 110, she started to feel better. We continued Fe for 6 months.'* (26 /A)

When patients had a history of similar symptoms in the past, or were frequent attenders, this influenced PCPs’ perceptions, so that they did not pay so much attention to these symptoms even where there were cancer risk factors:

*'The patient was well known to me after many years with many consultations because of different problems and complaints: muscular problems, chronic irritable colon, and anxiety. She was a cigarette smoker. She started coughing, and I was late to refer her to an X-ray of lungs. She had suffered for similar problems for many years, and was one of my patients with most consultations during the last years. I bagatellised* [played down] *her symptoms.'* (136 /V)

Sometimes other pre-existing or evolving health issues dominated PCPs’ thoughts, making it difficult to get a clear overview and act appropriately:

*'During extensive blood-tests for examination of fatigue in a 56-year-old woman I found an unspecific monoclonal gammopathia* [...] *because of stable values, I informed the patient and planned to control* [check] *the values twice a year the next years. However, during the following year, the patient developed poor mental health, and the follow-up of the values was forgotten in all the other follow-up throughout the following years.'* (83 /P)

PCPs also described how they could be influenced and distracted by people accompanying patients:

*'Her husband influenced me. I let him to influence my decision because there was a tension between them during the visit and the patient was ignoring her husband in an impolite manner.'* (44 /T)

### Patients’ hesitancy delayed the diagnosis

Sometimes primary care factors contributed to delayed presentations from their patients:

*'She didn't want to seek help because there were no GPs working there permanently,* […] *there was lack of them. She waited until there was GPs working permanently.'* (97/I)

In other cases, follow-up visits or tests were postponed by the patient because of practical difficulties or an unwillingness to be tested:

*'An old woman had cough for several months and came to my surgery for consultation. I advised for an X-ray but she had no way to go to the city at the time and she decided to postpone the examination.'* (14 /L)

Social problems related to patients themselves or their family members could distract patients from their own health problems. This could interfere with the PCPs’ investigation plans:

*'She lives alone with her daughter (who provides no type of support and causes social problems)* […] *she presents with a general deterioration and weight loss. Analytical tests (48 hours) are programmed and an appointment in 5 days to complete the anamnesis and physical examination. The patient doesn’t show up for the appointment.* [...] *Probably this is all due to the social problems and lack of support in her care.'* (53/D)

### System factors not facilitating timely diagnosis

Healthcare organisational factors could hamper the diagnostic process. Some PCPs described how they struggled with stress, work overload, and lack of time, where they felt pressured to act quickly as there were many other patients waiting for assessment, as in this example where a PCP explained why they had not followed cancer guidelines:

*'The patient is requiring his repeat prescription. You are constrained from time, because 15 more appointments* [are] *waiting for you with similar request that is* [for a] *repeat prescription.'* (68 /L)

Long waiting times for tests or specialist opinions and weaknesses in follow-up systems could contribute to delayed diagnosis. Limited PCP access to tests could interfere with the cancer diagnostic process:

*'… referred to ENT* [ear, nose, and throat]*, then waited 2 months for CT* [computerised tomography] *chest — diagnosed right-sided advanced lung cancer with paratracheal invasion; pathway issue, lack of access to diagnostics etc.'* (70/O)

Responders also described problems owing to a lack of continuity in patient care. When secondary and primary care were both involved, or when the PCP initiating the investigation was not available, there could be lack of clarity as to who should be responsible for the next step:

*'Skin biopsy was taken* [...] *The result were not checked until 6 months later. The doctor was on* [...] *summer holidays and also patient forgot to ask the PAD* [pathological and anatomical diagnosis] *diagnosis . It was found by accident when* [...] *doctor checking the old lab results.'* (118 /U)

Lack of PCP trainee experience and supervision could result in doctors managing symptoms, without thinking about what they could indicate:

*'60-year-old man came with low back pain. We made X-ray, blood tests. He had a fracture of L5. The pain was getting worse, and he died in a few months. I didn't have such experience. There were no programmes for prevention of cancer and any trainings for GP about cancers.'* (57/E)

### PCPs felt they had acted wrongly

Some PCPs thought that they had directly contributed to delayed cancer diagnoses; for example, because their history-taking or examination were inadequate. Some described ‘tunnel thinking’, when they were too focused on a single possible diagnosis early in the diagnostic process:

*'I diagnosed costal fractures without pneumothorax, clinically. I said it would take time to get well. She waited and waited, I told her to have patience. She did not get better. After months I took an X-ray – lung cancer with metastasis to bones, pathological fractures* […]. *One diagnosis made me not see the other.'* (30 /V)

PCPs gave examples of where follow-ups were forgotten or not planned, or when they had overlooked or did not act on abnormal test results:

*'I overlooked atypical leukocytes in an around 70–75y male patient.* [...] *Our haematology lab sheet is very long with about 30 indicators, some of which are quite often out of normal range (eg, RDW or monocytes). I just overlooked it.'* (148/Q)

Trusting reassuring opinions from specialists could stop PCPs searching for other possible explanations for their patients’ symptoms:

*'A woman in her 30s had dyspepsia and reflux.* [...] *I consulted with a gastroenterologist who deemed the patient too young to have serious stomach cancer risk.* [...] *Her young age and the gastroenterologist’s consultation put me at ease.'* (117 /F)

Some PCPs made the mistake of accepting patients’ ideas about a benign diagnosis. This stopped them investigating and safety netting:

*'An employee around 60 years at my former clinic came to me with a fatigue. She had been feeling tired from* [for] *half a year. She had been able to change her work content avoiding activities she felt increased the fatigue.* […] *Both I as a doctor and she as a patient zoomed in on "burn-out".'* (98/I).

### Problems with communicating adequately

PCPs mentioned a variety of issues relating to problems with communication with the patient, their relatives, or colleagues. They described the need on the one hand to be assertive and informative enough about the necessity for investigations and referrals, and on the other hand the wish to avoid worrying patients unnecessarily. Several PCPs saw this as their own failure in communication and engagement:

*'I advised for an X-ray but she had no way to go to the city at the time and she decided to postpone the examination. A month later she came again and suffer from haemoptysis.* […] *Patient was reluctant and I was not as persuasive as I ought to be in order to force her seeking evaluation from a specialist sooner.'* (14 /L)

*'*[On reflection] *I realise that I had a reluctance to refer as I thought this would cause alarm. I think this is subconscious, and had I considered cancer as a possibility I would, of course, have referred, but I wonder if my brain was steering me towards reassurance to avoid causing worry?'* (62 /H)

Unusual dynamics in the patient–doctor relationship could affect the diagnostic process:

*'His wife was a doctor and she often prescribed an antibiotic for infections herself* […] *Since he was also treated by his wife, he wasn’t consistently managed by me.'* (38 /K)

Responders also described poor communication, either from secondary care or from other healthcare providers:

*'The patient came with MRI* [magnetic resonance imaging] *report to her GP practice but the description of the spine MRI was very detailed and long and importantly there were no final conclusions or advice for clinicians regarding further steps. Two GPs saw the report but missed information written in small letters there might be metastases in the thoracic spine.'* (138 /G)

Colleagues with poor fluency in the local language could be a problem:

*'The radiologist was* [foreign] *and the report was difficult to understand because of language problems.'* (84/V)

## Discussion

### Summary

The results present European PCPs’ reflections on a complex, challenging task that they frequently face: making a timely diagnosis of cancer. Six themes were identified representing different layers of patient, PCP, and system-related factors that can interfere with the cancer diagnostic pathway. Communication challenges had an impact on all of the themes, and they were repeatedly described by the PCPs and expressed in various ways.

**Figure 1.**
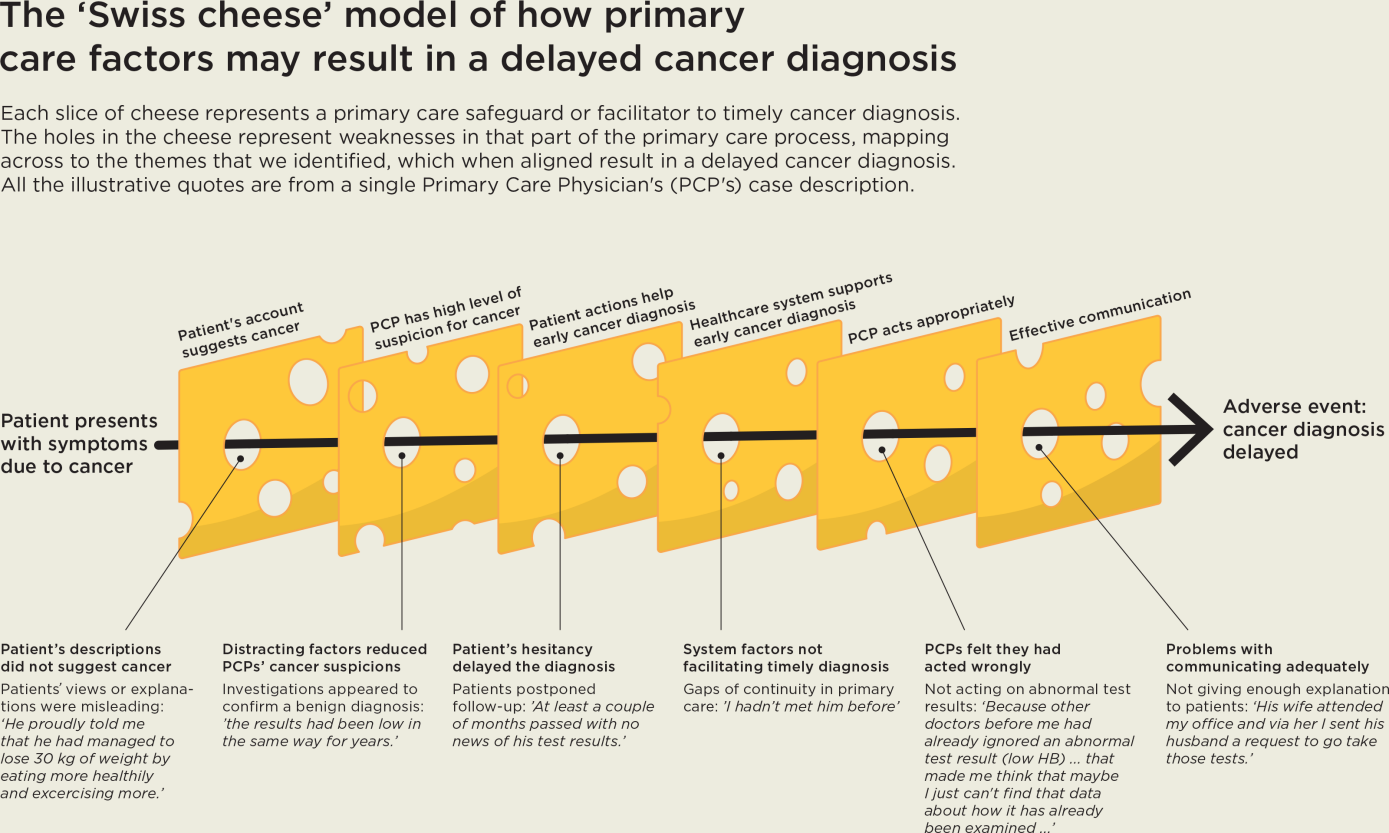
The 'Swiss cheese' model of how primary care factors may result in a delayed cancer diagnosis

The ‘Swiss cheese’ model of accident causation is a way of visualising how patient harm happens, based on a systems approach.^21^ In a complex healthcare system, errors are prevented by a series of defences, barriers, and safeguards, represented by slices of cheese. The holes in the slices represent unintended weaknesses in different parts of the system: when the holes in the slices align, a risk passes through all the holes, and this leads to a harmful failure of the system. The model is relevant to the findings, with the six themes mapping across to slices that represent safeguards or facilitators to timely cancer diagnosis, and the holes in them representing weaknesses in that part of the primary care process. Figure 1 gives an example of a pathway to a delayed cancer diagnosis that a participant reported in this study, with case-specific holes in all six theme-related safeguards or facilitators.

### Strengths and limitations

This is the first multinational study focusing on the experiences and reflections of PCPs who self-identified as having been slow to think of, or act on, a possible cancer diagnosis. It offers a comprehensive insight into the lessons to be learnt from participants’ cases in 23 European countries covering different healthcare systems, PCP demographics, work experiences and practice settings. A multinational team from the ÖRG carefully developed and piloted the questions, then performed the qualitative analysis, and so were able to consider the cultural and healthcare contexts of the participating countries. The large range of participating countries and the commonalities of PCPs’ experiences across those countries means that the identified themes are likely to be relevant to PCPs in other countries and healthcare systems.

The survey invited participants to share a single case, which could have prompted participants to select their most memorable cases rather than more common or typical ones. However, participants could have submitted several cases if they wanted to. Recall bias was possible, as some events had taken place several years earlier. Social desirability bias was also possible, as participants may have answered questions in a way that they thought would be viewed favourably by the researchers. Participants may have given incomplete descriptions, or there may have been response bias because of fear of litigation or complaint; however, it was made clear that participants’ responses would be anonymised. While the questionnaire language was English, which was not the native language of most participants, local study leads were asked to recruit only participants who would be likely to understand the survey questions. Some answers were translated by national teams, which may have resulted in missed nuances; however, many languages were represented in the analysis team. While data saturation was not assessed, the study had rich data from 158 PCPs, all their responses were analysed, and it is unlikely that new themes would have emerged with additional responders.

The data came from GPs in 23 European countries, each with their own cultural viewpoints, healthcare systems, and GP training processes.^22^ While it may therefore be that some of the identified themes are more commonly encountered in some countries than others, GPs from all the countries described issues that were encompassed by a wide range of the themes.

### Comparison with existing literature

The findings map across to those of other studies. One has confirmed that missed diagnostic opportunities can occur on several occasions, and can relate both to healthcare systems and to individuals.^7^ Other researchers have also found that system factors include lack of continuity in primary care and time pressures, as well as poor access to testing with long waiting times,^^23,24^^ and confirmed the findings of problems relating to gaps of continuity in primary care, fragmented care, and trainee supervision.^^7,23,24^^ In a 20-country European Delphi study, GPs came to a consensus that having quicker and easier communication with secondary care, shorter waiting times, and getting prompt advice from secondary care were essential for early cancer diagnosis.^^25^^

Other researchers have also described the findings that presentation of non-specific symptoms, the presence of other comorbidities, and symptomatic improvement, can be barriers to diagnosis.^^8,24,26,27^^ There is evidence that this can result in longer diagnostic intervals; for example, for colorectal cancer diagnosis in patients with mental health and gastrointestinal comorbidities.^28^ Some of the participants reported delays in diagnosis owing to poor history-taking or physical examination, and one author pointed out that these basic skills continue to be paramount.^^29^^

Some of the findings reflected those of another researcher, who reported that having an alternative working diagnosis, not reconsidering an initial diagnosis, and lack of follow-up were associated with long times to referral for patients with colorectal cancer.^26^ The present study's responders’ reports of inadequate plans for follow-up, as well as reassurance from normal test results, have also been reported by other researchers.^^8,30,31^^

### Implications for research and practice

This study offers a model of how a variety of factors can cause unintended weaknesses in the primary care diagnostic pathway, resulting in delayed cancer diagnoses. The model could be used to support the training of primary care clinicians, so that they are aware of the potential ‘holes in the Swiss cheese’ and how to avoid them.

The findings suggest that there needs to be cancer-specific training for PCPs, focusing on a systemic approach when organising tests and referrals, reviewing test results, and the use of follow-up and safety netting. There is also a need for ‘safe spaces’ for PCPs to discuss, share, and learn from their own experiences of delayed cancer diagnosis. Healthcare systems need to recognise the impacts on timeliness of cancer diagnosis of high PCP workload and poor continuity of PCP care, inadequate communication pathways between primary and secondary care, and poor access and waiting times for diagnostic tests and specialist opinions. Future studies should analyse how common the factors identified in this study are, quantify the effect that each of them has, and find out whether some are specific to particular healthcare systems.

In conclusion, European PCPs described cases where they considered that they had been slow to think of, or act on, a possible cancer diagnosis. The six overarching themes represent different layers of patient-, PCP-, and system-related factors that can interfere with the cancer diagnostic pathway, with communication challenges being common to all of them. The ‘Swiss cheese’ model of accident causation shows how the themes relate to each other. Addressing these issues should reduce morbidity and mortality in the small proportion of patients who have a considerable, avoidable delay in their cancer diagnosis.

## Data

To avoid the risk of identification of individual participants or patients, the datasets generated and analysed during the current study are not publicly available. However, they are available (with any identifying information redacted) from the corresponding author on reasonable request.
